# Overqualification Among Second-Generation Children of Immigrants in the Swedish Labour Market

**DOI:** 10.1007/s10680-024-09723-5

**Published:** 2024-11-26

**Authors:** Wooseong Kim

**Affiliations:** Demography Unit, Department of Sociology, Stockholm University, 106 91 Stockholm, Sweden

**Keywords:** Overqualification, Educational mismatch, Second generation, Immigrant integration, Human capital, Sweden

## Abstract

Research on the children of immigrants born in the host country (G2) consistently reveals disparities between their educational achievements and labour market outcomes compared to the majority population. This study provides new insights into understanding this disparity by examining patterns of overqualification—i.e., a downward educational mismatch—among the G2. Specifically, it explores 1) how overqualification patterns differ between the G2, foreign-born immigrants (G1), and the majority population and 2) how overqualification patterns vary across ten G2 ancestry groups compared to the majority population. Utilizing Swedish total population register data and linear probability models, this study estimates the probability of overqualification across different immigrant generations and ancestry groups, employing the Realised Matches method to measure overqualification. The results indicate that while G2 individuals have a lower probability of experiencing overqualification compared to G1, they face moderately higher probabilities of overqualification than the majority population—up to 19% higher. This disparity is particularly pronounced among G2 individuals with tertiary education and those of Iranian, Middle Eastern and North African, and Other Non-Western origins, with up to 39% higher probabilities. These findings suggest that G2 individuals, particularly those of non-Western origins, encounter significant challenges in translating their educational qualifications into commensurate employment within the Swedish labour market.

## Introduction

This study explores overqualification patterns among the second-generation (G2)––i.e., children of immigrants born in the host country. Overqualification refers to having educational qualifications exceeding those required to perform one’s job. Overqualified workers experience various labour market disadvantages, such as slower wage growth and a higher risk of unemployment than their underqualified or properly matched counterparts with similar educational attainment (Korpi & Tåhlin, 2009; Mavromaras et al., 2015). Thus, previous literature on educational mismatch has primarily focused on the determinants and consequences of overqualification.

Understanding the overqualification patterns of G2 individuals provides insight into their socio-economic integration in the host country. Although many migrant groups face greater socio-economic disadvantages than the majority population, i.e., natives with two native parents, the G2 is generally better off than their parents, which aligns with the new assimilation theory (Alba & Nee, 1997). In many Western European countries, G2 individuals often have similar or better educational outcomes than the majority population (Drouhot, 2024; Drouhot & Nee, 2019). Nonetheless, empirical evidence also shows that the G2 lags behind the majority population in labour market outcomes (Heath et al., 2008; OECD, 2017). This inequality between educational and labour market outcomes implies that many G2 individuals underutilise their human capital accumulated through formal education and fail to achieve occupational status commensurate with their educational achievements. This status inconsistency between educational and occupational achievements is an important yet often neglected dimension of the G2's socio-economic integration. Hence, this phenomenon warrants further investigation into their overqualification.

Furthermore, studying the overqualification patterns of G2 individuals provides a barometer of long-term ethnic inequalities related to the return on human capital investment. The G2 faces several barriers––e.g. hiring discrimination (Carlsson, 2010) and residential/school segregation (Hermansen et al., 2022; McAvay, 2018)—that make adequate education-occupation linkage challenging (Kracke & Klug, 2021; Rafferty, 2020). Importantly, these barriers are not equally distributed among G2 groups (Portes & Zhou, 1993; Zhou & Gonzales, 2019) and are likely to extend to their descendants, contributing to persistent ethnic stratification in occupational returns to education in the host society. Although previous research has already documented that foreign-born immigrants (G1), especially from non-Western countries, are disproportionately disadvantaged by overqualification (Joona et al., 2014; Nielsen, 2011), only a few studies have examined whether this disadvantage extends to G2 individuals (Belfi et al., 2022; Falcke et al., 2020; Khoudja, 2018; Larsen et al., 2018).

This study aims to describe overqualification patterns among the G2 and compare them to those among the majority population and the G1. Specifically, this paper investigates 1) how overqualification patterns differ between the G2, the G1, and the majority population and 2) how overqualification patterns vary across ten G2 ancestry groups compared to the majority population. To answer these questions, I used data from the total Swedish population register, covering all individuals registered in Sweden in 2016. Sweden provides a compelling case due to the growing number of G2 individuals entering the labour market with diverse ancestries. I used the *Realised Matches* (RM) method (Verdugo & Verdugo, 1989) to measure overqualification and linear probability models (LPM) to estimate the likelihood of overqualification across immigrant generations and ancestries.

This study contributes to the literature on qualification mismatch and immigrant labour market integration in three ways. First, this study is one of the first studies to investigate heterogeneity in overqualification across different ancestries. Given that the disadvantages associated with overqualification vary among G2 groups, it is crucial to examine this heterogeneity to understand the phenomenon comprehensively. Second, this study examines recent observations. Using recent data is essential for studying the labour market integration of the G2 in Sweden, as an increasing number of G2 individuals with diverse ancestries have entered the labour market only in recent years. This study contains a substantial number of G2 individuals of non-Western origin who were largely neglected in previous research (Dahlstedt, 2015). Third, this study uses detailed information on immigration records to overcome data issues that lead to the misclassification of immigrant generation (e.g., Fernández-Reino et al., 2018) and limitations in generalisability due to the selection into specific educational tracks (e.g., Falcke et al., 2020).

The main findings indicate substantial integration in occupational returns to education and underlying heterogeneity within the G2 across ancestry. The G2 shows a lower probability of overqualification than the G1, indicating integration in occupational returns to education. However, G2 individuals are still more likely to be overqualified than the majority population. Higher probabilities of overqualification are concentrated among G2 individuals of non-Western origins with tertiary education degrees. Having a native parent is associated with a lower probability of overqualification for most G2 groups except for G2 Finnish and Turkish groups. The observed heterogeneity in overqualification may reflect employer discrimination or other structural factors that contribute to significant challenges for G2 individuals in translating their educational qualifications into commensurate employment in the Swedish labour market.

## Literature Review

### Labour Market Integration of G2 Individuals

Unlike the G1, the G2 was born, raised, and educated in the host country and has more connections with the majority population. As a result, G2 individuals generally do not encounter the same challenges for labour market integration as their parents, such as lack of host country language skills and imperfect transferability of foreign qualifications (Alba & Foner, 2015). Likewise, their labour market outcomes are less determined by unobserved heterogeneity related to selection into immigration. A recent review on the socio-economic integration of the G2 in Western Europe identified a trend towards intergenerational socio-economic assimilation over time (Drouhot, 2024; Drouhot & Nee, 2019). The most significant convergence is observed in educational trajectories. Many G2 groups attain higher educational levels than their parents, and, in some cases, they surpass the educational attainment of their majority population peers (Borgen & Hermansen, 2023; Crul et al., 2012; Dollmann et al., 2023; Jonsson & Rudolphi, 2011). However, evidence of segmented assimilation (Portes & Zhou, 1993; Zhou, 1997) is apparent in the heterogeneous educational outcomes among G2 individuals (Alba & Foner, 2015; Baysu et al., 2018; Jackson et al., 2012).

The findings related to labour market outcomes of G2 individuals are nuanced. Although G2 individuals generally have better outcomes than G1, they still face barriers when transitioning from education to employment (Heath et al., 2008; OECD, 2017). Evidence from Sweden corroborates the findings from other Western European countries. G2 individuals of non-Western origin report higher unemployment rates than Nordic or Western-origin G2 groups and greater unemployment persistence (Aradhya et al., 2023). Nordic or Western-origin G2 individuals also report an overall convergence in earnings with the majority population, yet non-European-origin G2 individuals show relative disadvantages (Hammarstedt & Palme, 2012). Compared to earnings or employment, occupational attainment of the G2 has been discussed less in the literature. Although G2 individuals do not show systematic differences in occupational attainment once employed (Hermansen, 2013), overqualification is likely to be realised subtly, e.g., being hired in a position requiring a BA in Economics with an MA in Economics rather than explicitly, e.g., working as a taxi driver with an MA in Economics. Therefore, examining the G2’s overqualification patterns unveils nuanced inequalities in occupational attainment that previous research has not sufficiently addressed.

### Overqualification and Immigrant Populations and Their Descendants

#### Labour Supply- and Demand-Side Determinants of Overqualification

The major causes of educational mismatch can be divided into labour supply-side and demand-side determinants (see Ghignoni & Verashchagina (2014) for an overview). The labour supply-side explanations address individual heterogeneity, such as differences in human capital (Becker, 1964), educational quality (Ordine & Rose, 2009; Verhaest & Omey, 2006), individual preferences or constraints related to job search (Büchel & van Ham, 2003), and socio-economic background and social capital (Kracke & Klug, 2021). The labour demand-side explanations address the mechanisms that allow employers to intentionally hire overqualified candidates, such as employers’ job assignment (Thurow, 1976), institutional and structural changes in the labour market^1^ (Croce & Ghignoni, 2012; Di Pietro, 2002), and labour market discrimination (Pager & Pedulla, 2015; Rafferty, 2020). Within this framework, the determinants of overqualification specific to immigrant populations and their descendants are sorted together (see Table 1).

**Table 1:** Determinants of overqualification for the general population and migrants

	Supply-side	Demand-side
General	Individual heterogeneity in productivity, educational quality, and job search	Labour market institution Economic and technological changes Discrimination based on non-migrant-specific traits
Specific to G1	Language proficiency Transferability of skills and qualifications acquired before immigration	Discrimination based on foreign qualification and country of birth
Specific to G1 and G2	Ego’s job search networks	Discrimination based on ethnicity
Specific to G2	Parental SES background Social capital of migrant parents Friendship networks (peer effect on education/career)	

*Source*: Author’s own elaboration. G1 = First-generation immigrants, G2 = Second-generation children of immigrants, SES = Socio-economic status

The second row of Table 1 includes some key mechanisms shown to affect overqualification among the G1. However, many of them are not likely to contribute to the systematic differences in overqualification between the G2 and the majority population. First, lacking host country language proficiency increases G1 individuals’ overqualification risk (Aleksynska & Tritah, 2013; Budría & Martínez-de-Ibarreta, 2021). However, it is unlikely to explain the difference in overqualification among the G2 because G2 individuals and the majority population usually have similar language skills, especially among the highly educated. Second, the limited transferability of skills and foreign degrees contribute to the G1’s higher risks of overqualification (Chiswick & Miller, 2009; Lancee & Bol, 2017). However, these factors are unlikely to be a valid explanation for the overqualification of G2 individuals as they primarily receive education and acquire qualifications in the host country. Third, the G1 may face challenges securing adequate education-occupation alignment due to potential employer discrimination based on foreign qualifications (Damelang & Abraham, 2016) or country of birth (Jacobs et al., 2020). However, this demand-side factor is primarily related to the G1 and is not likely to cause systematic differences between the G2 and the majority population.

#### Determinants of Overqualification of the G2

The labour supply-side determinants related to potential systematic differences between the majority population and the G2 are co-ethnic environment, networks and differences in socio-economic background. The existing literature mainly discusses the influence of co-ethnic environments on job search networks. Two opposing arguments exist regarding the direction of the effect. On the one hand, informal job search based on co-ethnic networks may provide employment opportunities from labour market segments featuring higher concentrations of migrants, often referred to as employment niches, which are associated with a higher risk of overqualification (Kracke & Klug, 2021). On the other hand, co-ethnic networks may also increase employability and facilitate adequate job matches (Zwysen & Demireva, 2020). Importantly, co-ethnic environments are expected to contribute to heterogeneity in overqualification across ancestry groups since the influence of these environments relies on the size of co-ethnic groups and the amount of socio-economic resources co-ethnic groups possess (Bygren & Szulkin, 2010). The socio-economic background of migrant parents (Erdsiek, 2016; Zwysen & Longhi, 2018) may also contribute to a higher share of overqualified workers among the G2. Parental socio-economic background accounts for the disadvantages in the labour market transition that G2 individuals experience (Roth & Weißmann, 2022; Zwysen & Longhi, 2018). This determinant is also likely to contribute to differences among the G2 groups in the Swedish context because non-European immigrants generally show lower socio-economic status than the majority population or European immigrants (Wiesbrock, 2011).

The most crucial labour demand-side determinant of overqualification for the G2 is the employer’s statistical or taste-based discrimination (Aigner & Cain, 1977; Becker, 1971) against immigrant backgrounds or ethnic minorities. Concerning the overqualification of the G2, taste-based discrimination refers to an employer’s preference against a specific ethnicity or foreign background, and statistical discrimination refers to employers’ discriminatory behaviour based on existing stereotypes about foreign qualifications or foreign background due to lack of information about an individual’s actual qualifications and productivity (Leuven & Oosterbeek, 2011). Employer discrimination can contribute to a systematically higher risk of overqualification among the G2 than the majority population (Rafferty, 2020). Hiring discrimination against the G2 lowers their employability (see Lippens et al., 2022, for a systematic review) and increases their risks of long-term unemployment. To avoid long-term unemployment, the G2 may broaden their job search (Pager & Pedulla, 2015) and accept jobs that they are overqualified for. Furthermore, within employment, the G2 may be subject to allocative discrimination (Petersen & Saporta, 2004), i.e., employers allocate positions that require high skills or offer opportunities for promotion based on characteristics unrelated to individual productivity or qualifications.

It is reasonable to expect a heterogenous effect of discrimination on overqualification by ancestral origin because labour market discrimination is associated with visible characteristics, which are often associated with one’s ethnicity (Hersch, 2011), and perceived social and cultural distances between the majority population and migrants with different ancestry (Polek et al., 2010). For example, in the Swedish context, a G2 individual of non-Western origin is more prone to labour market discrimination than a G2 individual of Nordic or Western European origin (Carlsson, 2010).

When considering the heterogeneity within the G2 groups, it is also crucial to consider the distinct experiences of the 2.5 generation (G2.5) ––i.e., individuals with one native and one foreign-born parent. Exploring the differences in overqualification patterns between the G2 and the G2.5 provides an opportunity to investigate potential mechanisms related to the G2’s overqualification, such as job search networks and social capital. The G2.5 tends to exhibit higher degrees of social integration and better educational outcomes than their G2 counterparts (Kalmijn, 2015; Ramakrishnan, 2004). It may lead to frequent contact with their peers belonging to the majority population in their social networks (Kalmijn, 2015). However, this benefit may also be heterogeneous across ancestry due to differences in parental socio-economic status and stigmatisation of minority groups (Kalmijn, 2015; Tegunimataka, 2021).

Furthermore, the overqualification patterns among the G2 may differ by gender. Female employees may face higher levels of overqualification due to geographical mobility and time constraints (McGoldrick & Robst, 1996) and gender discrimination concerning promotion (Karakaya et al., 2007), another essential aspect of allocative discrimination. However, empirical findings generally do not find a significant gender gap in overqualification (Groot & Maassen Van Den Brink, 2000; Karakaya et al., 2007). In the Swedish context, G1 men are known to face a higher risk of overqualification than G1 women (Joona et al., 2014). Previous research also indicates that women with ethnic minority backgrounds may not necessarily face more severe labour market discrimination in Sweden (Arai et al., 2016). Therefore, the gender differences in overqualification patterns amongst the G2 are yet to be investigated.

#### Previous Research on Overqualification Among the G2

Compared to studies on the G1, a smaller volume of literature has investigated qualification mismatch among the G2 (Belfi et al., 2022; Dahlstedt, 2015; Falcke et al., 2020; Fernández-Reino et al., 2018; Khoudja, 2018; Larsen et al., 2018; Pecoraro, 2011; Tramosljanin, 2023; Weber et al., 2023). Most studies found that the G2 reports a lower risk of overqualification than the G1. However, there is no consensus on systematic differences between the G2 and the majority population. While several studies found similar overqualification patterns between the G2 and the majority population (Fernández-Reino et al., 2018; Khoudja, 2018; Larsen et al., 2018; Pecoraro, 2011; Weber et al., 2023), other studies highlighted the remaining gap between the two groups (Belfi et al., 2022; Dahlstedt, 2015; Falcke et al., 2020).

Moreover, despite the contributions of the existing literature, several gaps remain due to limitations in previous studies. For example, some findings are based on an unconventional definition of G2, including immigrants who arrived in the host country at a younger age (Fernández-Reino et al., 2018; Pecoraro, 2011). This approach may be problematic because those migrants still differ from the G2 due to previous experience of international migration at a young age, and some of them received education outside the host country. This study avoids this potential issue by utilising reliable information on the individual’s and parents’ country of birth and including enough number of G2 individuals from the total population register. Some other studies tend to concentrate on selected groups, such as recent graduates from professional universities (Belfi et al., 2022; Falcke et al., 2020). These findings are limited in generalisability because overqualification risks are distributed differently across the educational field (Ortiz & Kucel, 2008), and the G2 tend to choose academically more demanding educational routes (Borgen & Hermansen, 2023; Dollmann et al., 2023). This study solves this issue by including G2 individuals with various fields of study and educational attainment.

In the Swedish context, the only published study addressing the overqualification of the G2 is Dahlstedt (2015). He found that G2 men experience higher risks of overqualification than the majority Swedish men. However, Dahlstedt (2015) presents a significant limitation as it overlooks G2 groups of non-European origin, a group whose labour market disadvantages have been widely documented (Aradhya et al., 2023; Hammarstedt & Palme, 2012; Nordin & Rooth, 2009). Using recent data on G2 individuals from diverse origins, this study overcomes this limitation and further contributes to understanding the overqualification of G2 in the Swedish labour market.

Taken together, I anticipate that G2 in Sweden will generally exhibit a smaller gap in overqualification with the majority population compared to G1. This tendency is particularly expected for G2 individuals of Nordic or Western origins. However, G2 individuals of non-Western origins may still face a higher probability of overqualification. Furthermore, the probability of overqualification is expected to be lower among the G2.5 individuals than G2 individuals, although the extent of this variance may depend on ancestral background.

## Data and Methods

### Data

I used the collection of Swedish register data collected by national public authorities and provided by Statistics Sweden (SCB) for a broader research project, the *Migrant Trajectories.*^2^ SCB gave users access to the data through an online microdata access system (MONA). The dataset included individuals registered in Sweden from 1968 to 2017. The registered individuals were given a unique personal identity number (*personnummer*), which SCB anonymised and made available in the study data. Researchers could link children to their parents using this anonymised personal identity number in the Swedish Multi-Generation Register.

**Fig. 1:**
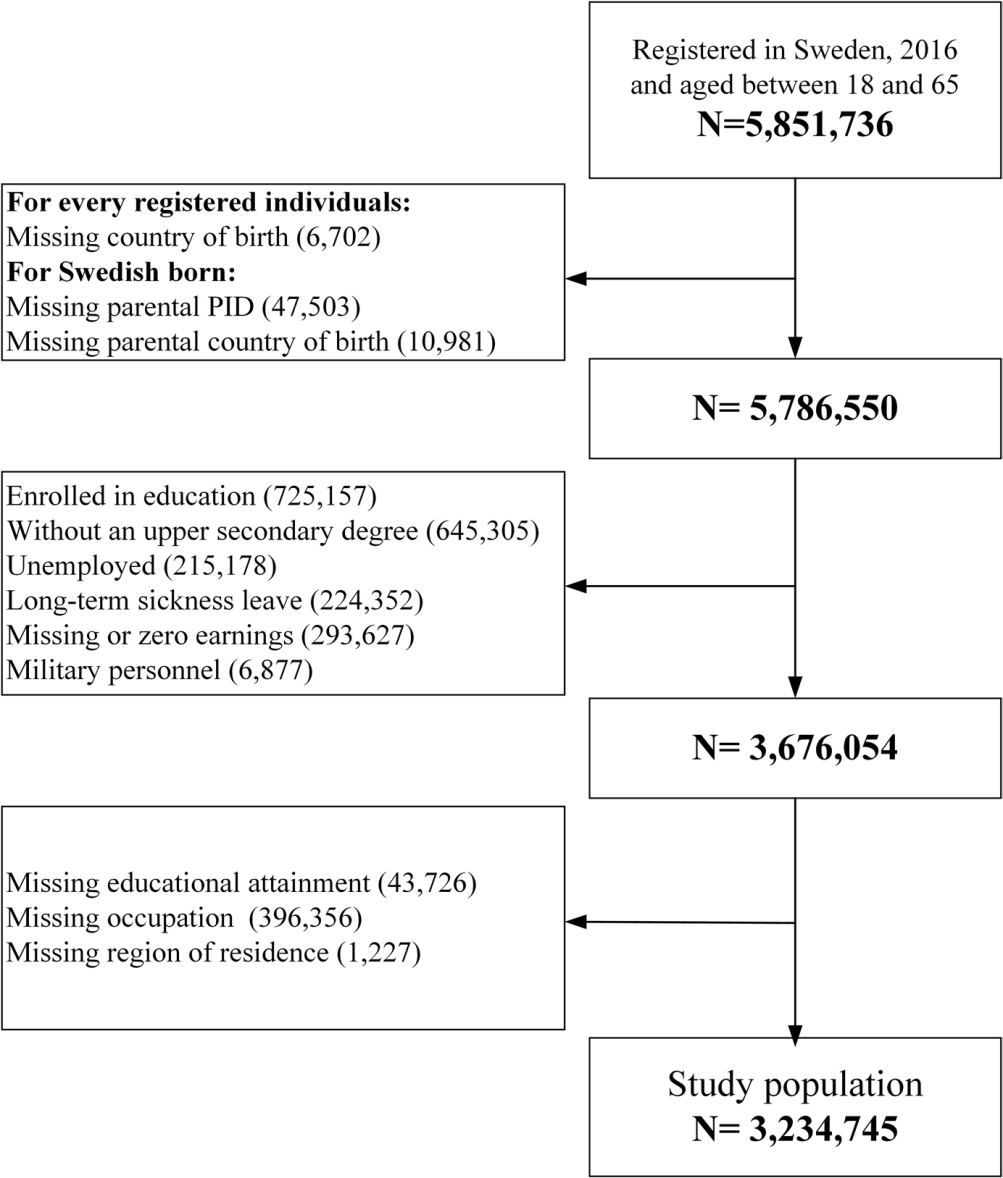
Flow chart of the selection of the study population

Figure 1 presents a flow chart diagram of the study population selected. The study population included all individuals between 18 and 65 years old, registered in Sweden in 2016, employed (i.e., having non-zero labour income, having fewer than 90 days of registered unemployment (Aradhya et al., 2023), not in education, and not in long-term sickness leave), and with at least an upper secondary education degree. Similar to the previous studies focusing on university graduates or postgraduates (Banerjee et al., 2019; Jacobs et al., 2020; Verhaest & Van Der Velden, 2013), I excluded those without upper secondary degrees because they are unlikely to be overqualified, irrespective of the overqualification measure used. This study followed a complete case analysis design—i.e., any observations with missing variables required to measure qualification mismatch or immigrant generation/ancestry were excluded. Consequently, the study population included 3,234,745 observations.

### Variable

#### Education and Occupation Measure

I measured educational attainment using annually recorded information on the highest completed educational degree from the Educational Register (UREG, *utbildningsregistret*). This register, which uses the Swedish educational classification 2000 (*Svensk utbildningsnomenklatur*, SUN 2000), includes information on the level and field of the individual’s highest educational degree registered. SUN 2000 is based on the International Standard Classification of Education 97 (ISCED 97) with some adaptations to align with the Swedish context. The highest completed degree refers to the end of the Spring semester of a given year (Statistics Sweden, 2019). Based on this information, the years of schooling are approximated to operationalise the qualification mismatch measure. I measured occupation using data from the Swedish Occupational Register (*yrkesregistret*), which provides information about an individual’s occupation on an annual basis. The Swedish Occupational Register is available from 2001 and uses the four-digit Swedish Standard Classification of Occupations (SSYK) to identify the skill level and degree of specialisation associated with an individual’s registered occupation. SSYK is based on the International Classification of Occupations (ISCO) 88 (2001–2013) and ISCO-08 (2014–2016). Most occupation data covering private and governmental sectors refer to data collected annually on September 1. Occupation data for service on county councils, municipality offices, and the Church refer to data collected annually on November 1 (Statistics Sweden, 2011).

#### Overqualification Measure

Although there has been extensive methodological debate on how to measure qualification mismatch, there has been no consensus on the most suitable indicator among three commonly used approaches: Job Analysis (JA), Worker Self-Assessment (WA), and Realised Matches (See Capsada-Munesh (2019) for discussion). In this study, I chose the RM method, first proposed by Clogg and Shockey (1984) and further developed by Verdugo and Verdugo (1989). The JA method is an objective and normative approach relying on a predetermined alignment between education and professions developed by job experts (Capsada-Munsech, 2019). Although the JA method objectively measures qualification mismatch (Hartog, 2000), constructing sufficiently detailed comparisons between educational and occupational qualifications requires massive effort and time. Therefore, it is prone to be outdated and affected by credential inflation (Capsada-Munsech, 2019), which makes it more vulnerable to misclassification. The WA method is a subjective measure which relies on the worker’s self-reported assessment of the required job qualifications (Hartog, 2000). This method is straightforward as it uses simple questions; however, it is prone to social desirability bias and likely to be influenced by other job characteristics such as wage or employment security (Capsada-Munsech, 2019).

On the other hand, the RM approach is suitable for exploring an individual’s status (relative) compared to others in the same occupation (Capsada-Munsech, 2019). Ample observations from large-scale population registry data enable the construction of more reliable cut-offs than survey data (Joona et al., 2014; Larsen et al., 2018) because the registry data provide detailed occupation classifications and are less likely to be influenced by outliers. In addition, the RM method is flexible enough to consider compositional factors, such as age groups (e.g., Wanner et al., 2021). However, this method is limited as it assumes that all skills are obtained through schooling and overlooks variations in educational requirements across jobs within the same occupation (Capsada-Munsech, 2019).

In this study, I used the modal value of years of schooling within an occupation block, defined by the four-digit SSYK code and calculated by each age group (< 30,30–39,40–49, ≥ 50). Additional standardisation by age group was introduced to consider the potential increase in qualifications due to the expansion of higher education in Sweden. I used all four digits of SSYK to account for differences across industries. Individuals in the analytic sample were defined as overqualified if their years of schooling were higher than the modal value. Thus, the reference group consisted of adequately matched and underqualified individuals. If the mode was not a unique value, a worker was identified as overeducated when the worker’s years of schooling exceeded the highest number of modal years of schooling. I calculated modal values only using Swedish-born workers to prevent the immigrant-native segregation in occupations from distorting the distribution of years of schooling. I conducted the same analyses for robustness checks using the mean-based method with a one-standard-deviation threshold. Although the estimated share of overqualification was lower with the mean-based methods, the point estimates from regression models were comparable to the mode-based method (see column (3) in Table 8 and column (3) in Table 9 in the Appendix for more information).

#### Immigrant Generation and Ancestry

The immigrant generation was identified using country of birth and parental country of birth. The reference group, the majority population, was defined as individuals born in Sweden with two Swedish-born parents. First-generation immigrants were defined as individuals born outside Sweden. Second-generation children of immigrants were defined as individuals born in Sweden with at least one foreign-born parent. For further analysis, the G2 with one native and one foreign-born parent was classified as a separate category, G2.5. Ancestry was assigned to Swedish-born individuals and defined as the father’s country of birth.^3^ The reason for following the father’s ancestry is that it is more common to use the father’s surname in Sweden, which is associated with ethnic identity in the Swedish labour market (Bursell, 2012, 2014). I also conducted analyses with an alternative definition of ancestry using the mother’s country of birth, and the results indicate that the differences in point estimates are minor (see column (4) in Table 9 in the Appendix). I distinguished between 11 ancestries: Sweden, Finland, Other Nordic, Other Western (including both European and non-European Western countries, such as the United States and Australia), Eastern Europe, Former Yugoslavia, Southern Europe (excluding former Yugoslavia), the Middle East and North Africa (MENA, excluding Iran), Iran, Turkey, and Other Non-Western (see Table 11 in the Appendix for more information). Although the ancestry classification mostly followed broader geographical areas, I separated particular origin groups with unique integration experiences in Sweden. For example, Finnish migrants have a long immigration history in Sweden and are relatively well integrated into the labour market. Furthermore, a substantial share of Finnish migrants immigrated from a Swedish-speaking ethnolinguistic minority group in Finland (Saarela & Scott, 2017). Another example is Iranian migrants. Most arrived in Sweden as refugees, and although their children outperform the majority population in post-secondary education attainment, this advantage does not necessarily translate into better socio-economic outcomes (Harber-Aschan et al., 2022).

#### Control Variables

This study adjusted for essential compositional factors. First, age and the square term of age were included because the risk of overqualification is associated with age due to correlation with work experience (Joona et al., 2014), and the G2 groups may have different age distributions compared to the majority population. Second, I included the region of residence (rural/suburban/urban) as the labour market size and geographical mobility affect job-matching behaviour (Büchel & van Ham, 2003). This variable was based on the Demographic Statistical Areas (DeSO) categorisation provided by SCB (Statistics Sweden, 2024). As the main aim of this study was to describe the total association between overqualification and immigrant generation and ancestry rather than providing any causal inference, I did not include factors that mediate the total association, such as parental socio-economic background, field of study, or industry. Tables 4 and 5 in the Appendix summarise the descriptive statistics of the control variables by immigrant generation and ancestry.

### Analytic Strategy

I conducted statistical analyses in four steps. First, I estimated the percentage of overqualified employees by immigrant generation and ancestry using the mode-based and mean-based methods separately for men and women. In the Appendix, I included the results from observations in 2013 to see to what extent the change in SSYK between 2013 and 2014 affected the results. Second, I estimated the association between immigrant generation and overqualification probabilities by fitting LPMs adjusted for control variables. The benchmark model (Model 1) was estimated without any interaction terms. I also estimated a model with three-way interaction terms between immigrant generation, gender (men/women), and educational attainment (upper secondary/tertiary) to see if the associations differ by gender and education level (Model 2). For a robustness check, I conducted the same model as Model 2 using the mean-based measure of overqualification (Model 3). Third, I estimated the association between ancestry and overqualification by fitting LPMs. Like in the second step, I first estimated the benchmark model adjusted for age, age squared, region, and the G2.5 status without interaction terms (Model 4). I also estimated a model with the three-way interaction terms between ancestry, gender, and educational attainment (Model 5). For a robustness check, I conducted the same analysis as Model 5 using the mean-based method (Model 6) and an alternative classification of the ancestry group (Model 7). Finally, as a further analysis, I constructed a new categorical variable that contains all combinations of ancestry groups and G2.5 status and estimated differences in overqualification probabilities to see if the gaps between the G2 and the G2.5 groups show heterogeneous patterns, with and without the three-way interaction term between ancestry groups, gender, and educational attainment (Model 8 and Model 9). I only included Swedish-born individuals (excluding the G1) for the third and fourth steps.

I chose LPMs over nonlinear models such as the logit specification because this study contains empirical exercises involving direct comparisons of point estimates across models. LPMs also enable a more straightforward interpretation of interaction terms. The odds ratio estimated by logistic regressions is unsuitable for direct comparisons (Mood, 2010), and average marginal effects obtained from logit models are comparable to unstandardised coefficients from the LPMs (Angrist & Pischke, 2009). Because the study data contain many observations and the models are estimated using heteroskedasticity robust standard errors, the LPM is a consistent estimator even for binary outcomes (Angrist & Pischke, 2009; Hellevik, 2009).

## Results

### Descriptive Findings

**Fig. 2:**
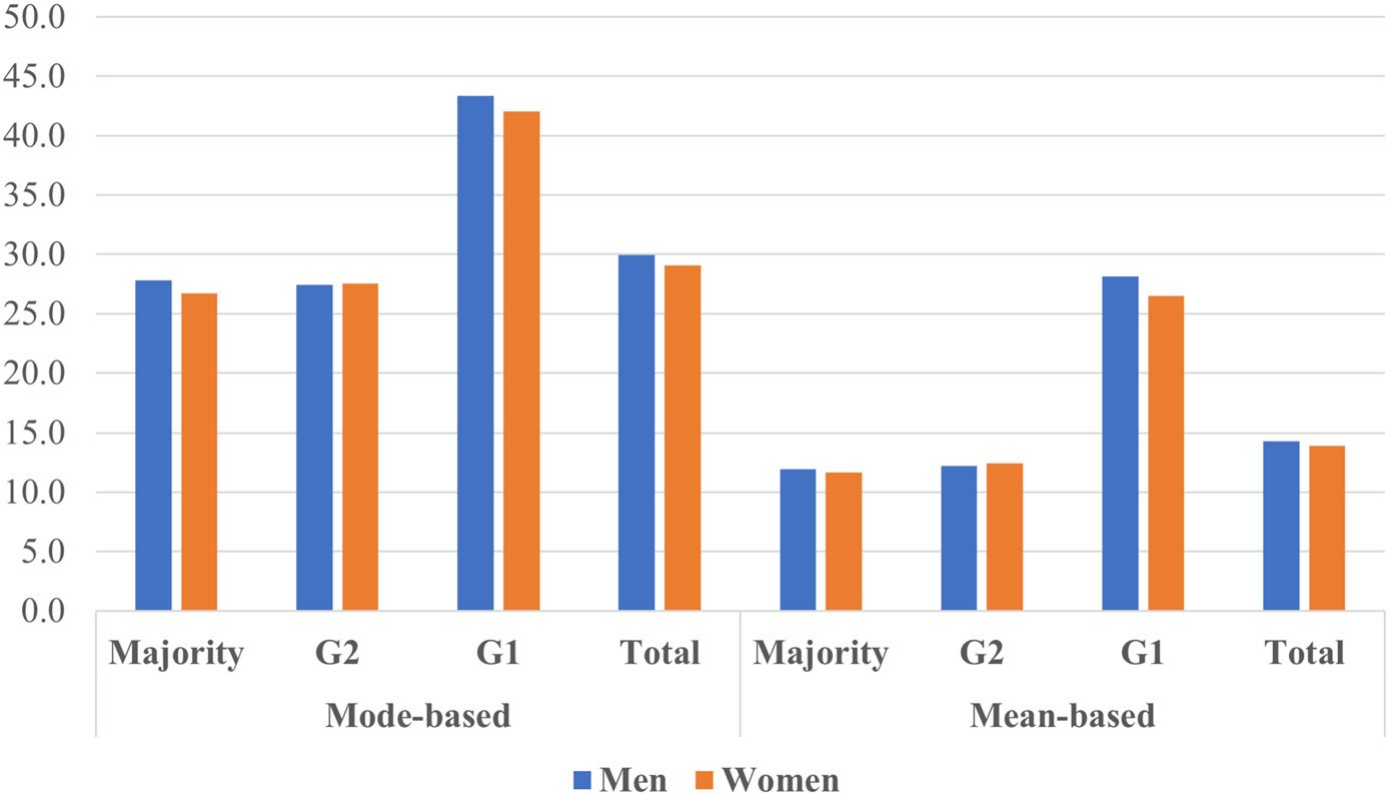
Prevalence of overqualification (%) by immigrant generation and gender, using the mode-based and mean-based methods (see Table 6)

Figure 2 presents the share of overqualification in the study population by immigrant generation and gender (See Table 6 in the Appendix). Overall, the descriptive findings indicate that the share of the overqualified among the G2 is comparable to that of the majority population. At the same time, the G1 stands out with the largest share of overqualified workers. According to the results calculated with the mode-based method, the proportion of overqualified employees among the majority Swedish population was 27.8% for men and 26.7% for women. G2 men and women showed similar shares (27.5%) compared to their majority population counterparts. Meanwhile, G1 men (43.3%) and G1 women (42.1%) reported substantially higher shares of overqualified employees than other groups. The results by the mean-based method show a lower proportion of overqualified employees for all groups. Both results show a relatively small gap between the G2 and the majority population and substantially higher levels of overqualification among the G1. For a robustness check, I calculated the share of overqualified employees using the observations from 2013. Although there were minor differences in prevalence, the results were similar between 2013 and 2016 (see Table 6).

**Fig. 3:**
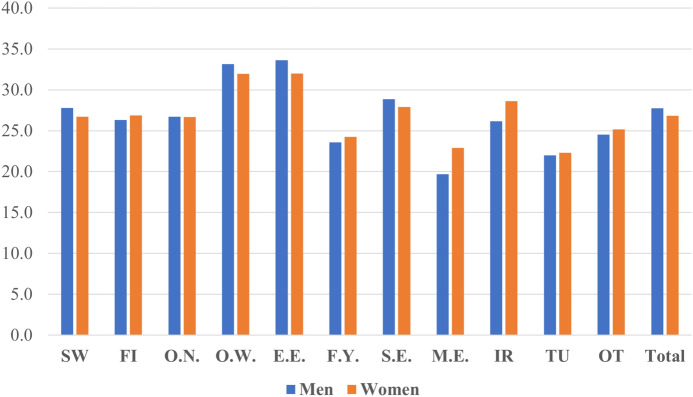
Prevalence of overqualification (%) among Swedish-born individuals by ancestry and gender, using the mode-based method (see Table 7). *Note*: SW = Sweden, FI = Finland, O.N. = Other Nordic, O.W. = Other Western, E.E. = Eastern Europe, F.Y. = Former Yugoslavia, S.E. = Southern Europe, MENA = Middle East and North Africa, IR = Iran, TU = Turkey, OT = Other Non-Western

Figure 3 presents the share of overqualification among Swedish-born individuals by ancestry and gender, calculated using the mode-based methods (see Table 7 in the Appendix for the results from the mean-based method). It reveals the heterogeneity across ancestry groups, which was not visible in Fig. 2. Among men, G2 Eastern European (33.7%), Other-Western (33.2%), and Southern European (28.9%) men displayed higher shares of overqualified employees compared to the majority Swedish men (27.8%). Meanwhile, G2 MENA (19.7%), Turkish (22.0%), Former Yugoslavian (23.6%), and Other Non-Western (24.6%) men showed lower shares of overqualified employees compared to the reference group. Among women, the overall pattern is similar to men with two notable differences: first, G2 Iranian women (28.5%) report a 1.8 percentage point (pp) higher share of overqualified employees compared to the majority Swedish women (26.7%). Second, while the share of overqualified employees was lower for women among the majority population, the opposite is true for many G2 groups. Table 7 in the Appendix shows the results based on the mean-based method from 2013. Similar to the findings from Table 6, the shares calculated with the mean-based method tend to be lower, while the rankings among ancestry groups are mainly unchanged. The results were similar between 2013 and 2016.

The descriptive findings suggest that the G2 shows integration in occupational returns to education compared to the G1 in the study population. However, the overall integration patterns seen in Fig. 2 are not evenly distributed across ancestry groups. While G2 individuals of Nordic, Former Yugoslavian, MENA, and Turkish ancestry show similar or lower shares of overqualified employees, G2 Other Western, Eastern European, Southern European and Iranian groups showed somewhat higher proportions of overqualified employees than the majority Swedish population.

### Differences in Overqualification by Immigrant Generation

Table 8 in the Appendix presents the estimated differences in probabilities of overqualification from LPMs. The estimates in column (1) are from Model 1 without interaction terms, and the estimates in column (2) are from Model 2 with three-way interaction terms between immigrant status, gender, and educational attainment. The results in column (1) confirm integration in occupational returns to education among the G2 and the remaining gap between the G2 and the majority population. The G1 ($\beta _{{G1}}$ = 0.129) shows a higher probability than the G2 ($\beta _{{G2}}$= 0.025) and the G2.5 (${\beta }_{G2.5}$ = 0.010). Although the G2 and the G2.5 present a substantially lower probability of being overqualified than the G1, the point estimates for these groups are still larger than zero, ranging from 1 to 2.5 pp. The result in column (2) shows that G2.5 women, G2 men, and G2 women groups with tertiary education degrees mainly contribute to this remaining gap. The interaction term between immigrant generation and educational levels and the three-way interaction terms are statistically significant at the 5% level.

**Fig. 4:**
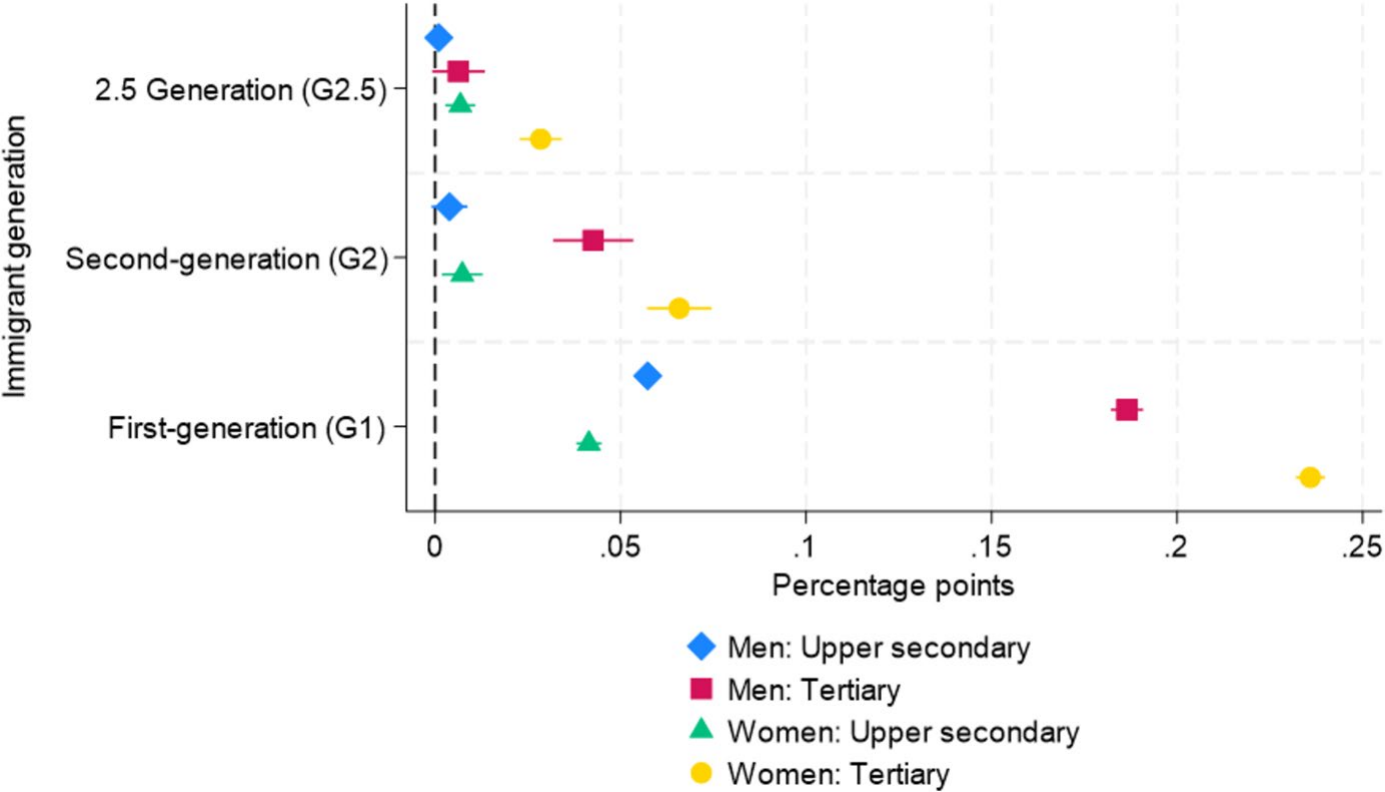
Estimated differences in overqualification probability across immigrant generation groups by gender and educational (See column (2) in Table 8 in the Appendix). *Note*: The point estimates are presented with 99% confidence intervals. The model is controlled for age, age squared, and region

Based on the estimation results from Model 2 in Table 8, Fig. 4 displays the estimated differences in probabilities of overqualification across immigrant generations by gender and education level. The G1 is more likely to be overqualified than the G2 and the G2.5, regardless of gender and education level. Among the tertiary educated, G2 men and women display 4.3 (${\beta }_{G2 men}$: 0.043) and 6.6 (${\beta }_{G2 women}$: 0.066) pp higher probabilities of overqualification than the majority Swedish population. G1 men and women with tertiary education degrees display 18.7 (${\beta }_{G1 men}$: 0.187) and 23.6 (${\beta }_{G1 women}$: 0.236) pp higher probabilities of overqualification than the reference group. The estimated coefficients for G2.5, G2, and G1 men and women with tertiary education degrees correspond to 1%, 9%, and 38% higher probabilities of overqualification for men and 8%, 19%, and 67% higher probabilities of overqualification for women, respectively. For example, this result means that for every 100 majority Swedish women, there are, on average, 119 G2 women and 167 G1 women amongst overqualified tertiary-educated women.

### Differences in Overqualification by Ancestry

Table 2 presents the estimated differences in probabilities of overqualification from LPMs (see Table 9 in the appendix). The estimates in column (1) are from Model 4 without interaction terms, and the estimates in column (2) are from Model 5 with three-way interaction terms between ancestry, gender, and educational attainment. The result in column (1) shows substantial differences in probabilities of overqualification across ancestry groups. For instance, G2 Iranian (${\beta }_{Iranian}$ = 0.071), Eastern European ($\beta _{{Eastern\;European}}$ = 0.042), Other Western (${\beta }_{Other\;Western}$ = 0.040), and Other Non-Western (${\beta }_{Other\;Non-Western}$ = 0.039) report higher probabilities than the reference group, i.e., the majority Swedish population, ranging between 3.9 and 7.1 pp. Having one native parent (G2.5) is associated with a 1.2 pp lower probability of overqualification.

**Table 2:** The selection of point estimates of the differences in the probability of overqualification across ancestry groups from LPMs (see Table 9 for full results)

	(1)	(2)
	Model4	Model5
*Ancestry*		
*(Reference* = *Sweden)*		
Finland	0.010***	0.001
	(0.002)	(0.002)
Other Nordic	0.008**	0.001
	(0.002)	(0.003)
Other Western	0.040***	0.048***
	(0.003)	(0.004)
East-Eu	0.042***	0.044***
	(0.003)	(0.004)
Former Yugoslavia	0.018***	− 0.003
	(0.003)	(0.004)
South-Eu	0.021***	0.015**
	(0.004)	(0.006)
MENA	0.027***	− 0.010**
	(0.003)	(0.004)
Iran	0.071***	0.025**
	(0.006)	(0.008)
Turkey	0.010*	− 0.026***
	(0.004)	(0.005)
Other	0.039***	− 0.002
	(0.003)	(0.004)
*G2.5*		
*(Reference* = *No)*		
Yes	− 0.012***	− 0.013***
	(0.002)	(0.002)
Gender		
(Reference = Men)		
Women	− 0.049***	0.001*
	(0.001)	(0.001)
*Educational attainment*		
*(Reference* = *Upper secondary)*		
Tertiary	0.231***	0.303***
	(0.001)	(0.001)
*Interaction terms*		
*Ancestry X Gender*		
Finland & Women		0.005
		(0.003)
Other Nordic & Women		0.009*
		(0.004)
Other Western & Women		0.004
		(0.005)
East-Eu & Women		− 0.007
		(0.006)
Former Yugoslavia & Women		− 0.005
		(0.006)
South-Eu & Women		− 0.005
		(0.009)
MENA & Women		0.025***
		(0.006)
Iran & Women		0.029*
		(0.013)
Turkey & Women		− 0.001
		(0.008)
Other & Women		0.015**
		(0.005)
*Ancestry X Educational attainment*		
Finland & Tertiary		0.019***
		(0.004)
Other Nordic & Tertiary		0.008
		(0.007)
Other Western & Tertiary		− 0.039***
		(0.006)
East-Eu & Tertiary		− 0.022**
		(0.008)
Former Yugoslavia & Tertiary		0.052***
		(0.010)
South-Eu & Tertiary		− 0.005
		(0.012)
MENA & Tertiary		0.055***
		(0.012)
Iran & Tertiary		0.060**
		(0.019)
Turkey & Tertiary		0.102***
		(0.014)
Other & Tertiary		0.071***
		(0.009)
*Ancestry X Gender X Educational attainment*		
Women & Tertiary		− 0.137***
		(0.001)
Finland & Women & Tertiary		0.006
		(0.006)
Other Nordic & Women & Tertiary		− 0.001
		(0.009)
Other Western & Women & Tertiary		0.031***
		(0.009)
East-Eu & Women & Tertiary		0.041***
		(0.010)
Former Yugoslavia & Women & Tertiary		0.038**
		(0.014)
South-Eu & Women & Tertiary		0.043**
		(0.016)
MENA & Women & Tertiary		0.031*
		(0.015)
Iran & Women & Tertiary		0.024
		(0.027)
Turkey & Women & Tertiary		− 0.002
		(0.019)
Other & Women & Tertiary		0.019
		(0.012)
Observations	2,763,905	2,763,905
Adjusted R-squared	0.085	0.090

East-Eu = Eastern European. Suth-Eu = Southern European. Other = Other Non-Western. Standard errors in parentheses

*p < 0.05, **p < 0.01, ***p < 0.001

The results from Model 5 reveal that, for most ancestry groups, these associations vary by gender and educational level. The point estimates for the interaction terms between ancestry and gender are generally small, indicating minor gender differences among upper secondary graduates. However, there are notable exceptions: women from G2 MENA, Iranian, and Other Non-Western groups show positive and statistically significant interaction terms at the 5% level.

Regarding the interaction between ancestry and educational attainment, the point estimates indicate that, for many G2 origin groups, individuals with tertiary education have additionally higher probabilities of being overqualified compared to their majority population peers with the same level of education. Notably, the interaction terms between ancestry and tertiary degrees are positive and statistically significant at the 5% level, except for G2 Other Nordic, Other Western, Eastern European, and Southern European groups.

Moreover, the point estimates of the three-way interaction terms (ancestry, gender, and educational level) are greater than zero and statistically significant at the 5% level for several origin groups, including G2 Other Western, Eastern European, Former Yugoslavian, Southern European, and MENA women. These findings suggest that the overqualification gap between G2 individuals with tertiary education and their majority population counterparts is more pronounced among women from these origin groups compared to men.

**Fig. 5:**
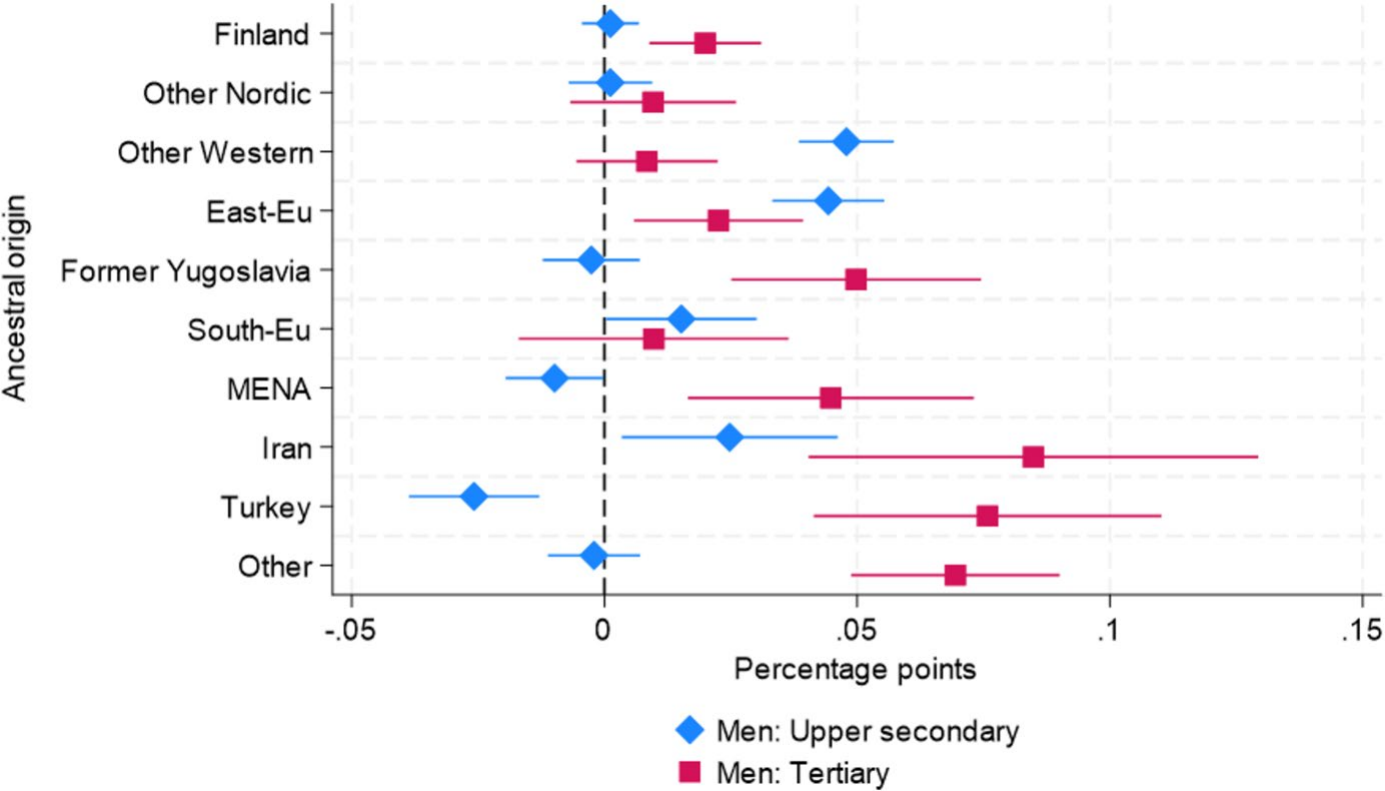
Estimated differences in overqualification probability across ancestry groups for men by educational level. *Note*: The point estimates are presented with 99% confidence intervals. The model is controlled for age, age squared, region, and G2.5 status

**Fig. 6:**
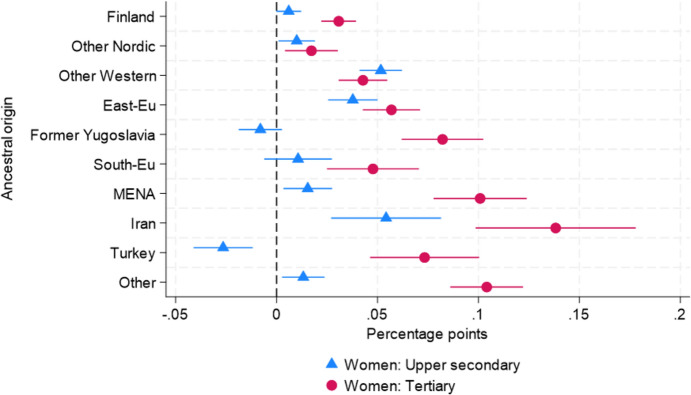
Estimated differences in overqualification probability across ancestry groups for women by educational level. *Note*: The point estimates are presented with 99% confidence intervals. The model is controlled for age, age squared, region, and G2.5 status

Based on the estimation results from Model 5, Figs. 5 and 6 display the probabilities of overqualification across ancestry groups by gender and educational attainment. As shown in Table 2, the two Figures display notable differences in probabilities of overqualification between upper secondary graduates and tertiary education graduates among G2 individuals. In Fig. 5 G2 men with upper secondary are divided into two groups: those who report a lower or similar probability of overqualification than the majority Swedish men, such as G2 Finnish, Other Nordic, Former Yugoslavian, MENA, Turkish, and Other Non-Western groups; and those with a higher probability of overqualification than the majority Swedish men, such as G2 Other Western, Eastern European, South European, and Iranian groups. The probabilities for the latter group range between 1.5 and 4.8 pp. Most G2 men with tertiary education degrees report higher probabilities than the majority Swedish male counterparts except for G2 Other Nordic, Other Western and Southern European men. They report probabilities of overqualification ranging between 0.8 (G2 Other Western) and 8.5 pp (G2 Iranian). For instance, the point estimate for G2 Iranian men with tertiary education degrees corresponds to a 17% higher probability of overqualification than the majority Swedish men.

Figure 6 shows similar overqualification gaps between upper secondary and tertiary education graduates among G2 women groups, with a few exceptions. Contrary to G2 Other Western and Southern European men, G2 Other Western and Southern European women with tertiary education degrees show positive probabilities of overqualification, and the levels are similar or even higher than their counterparts. Also, as shown in Table 2, the probability gap between upper secondary and tertiary education graduates is larger among G2 Iranian women than among G2 Iranian men. The probabilities of overqualification among G2 women range between 1.7 (G2 Other Nordic) and 13.8 pp (G2 Iranian). For instance, the point estimate for G2 Iranian women with tertiary education degrees corresponds to a 39% higher probability of overqualification than the majority Swedish women.

Table 3 compares probabilities of overqualification between G2 and G2.5 for each ancestry group by gender and education level (see Table 10 in the Appendix). The point estimates are from the regression model adjusted for age, age squared and region, and including three-way interaction terms between ancestry group, gender, and educational attainment as shown in column (2) in Table 10 in the Appendix (Model 9). In general, G2.5 is associated with a lower probability of overqualification except for two groups: G2 Finnish women with tertiary education degrees and G2 Turkish women with upper secondary degrees. For example, G2.5 Iranian men with tertiary education degrees show an 8.5 pp lower probability of overqualification than G2 Iranian men with the same degrees. Furthermore, the point estimate for G2.5 Iranian men is not statistically significantly higher at the 5% level compared to the reference group. Similarly, G2.5 MENA men, Turkish women, and Other Nordic women groups also show similar probabilities of overqualification compared to the reference groups, i.e., the majority Swedish men and women. Meanwhile, G2.5 Finnish women with tertiary education degrees report a 1.3 pp higher probability of overqualification than their G2 Finnish counterparts, and G2.5 Turkish women with upper secondary education also report a 2.1 pp higher probability than their G2 Turkish counterparts. Interestingly, this reversal between the G2 and the G2.5 was not observed among men, i.e., having one native parent is associated with a lower or similar probability of overqualification for all male ancestry groups. One should note that not all gaps between the G2 and G2.5 in Table 3 are statistically significant, as the p-values only indicate whether differences from the reference group (the majority population) are statistically significant.

**Table 3:** Estimated differences in the probability of overqualification between the G2 and the G2.5 by ancestry groups, gender, and educational attainment from LPM (see Table 10 in the Appendix)

	(1)	(2)	(3)	(4)
	Men upper sec	Men tertiary	Women upper sec	Women tertiary
*Ancestry group*				
*(Reference* = *Sweden)*				
Finland G2	− 0.005	0.016*	− 0.006	0.012*
	(0.003)	(0.008)	(0.004)	(0.006)
Finland G2.5	− 0.009***	0.008	− 0.001	0.025***
	(0.002)	(0.005)	(0.002)	(0.004)
Other Nordic G2	0.007	− 0.004	0.020*	0.060***
	(0.008)	(0.018)	(0.009)	(0.014)
Other Nordic G2.5	− 0.012***	− 0.001	− 0.004	− 0.001
	(0.003)	(0.007)	(0.003)	(0.005)
Other Western G2	0.055***	0.020	0.055***	0.042**
	(0.009)	(0.015)	(0.011)	(0.013)
Other Western G2.5	0.034***	− 0.006	0.038***	0.030***
	(0.004)	(0.006)	(0.004)	(0.005)
Eastern Europe G2	0.051***	0.020	0.048***	0.068***
	(0.007)	(0.011)	(0.008)	(0.009)
Eustern Europe G2.5	0.028***	0.011	0.020***	0.039***
	(0.005)	(0.008)	(0.005)	(0.007)
Former Yugoslavia G2	− 0.001	0.056***	− 0.012*	0.088***
	(0.005)	(0.012)	(0.005)	(0.010)
Former Yugoslavia G2.5	− 0.018**	0.027	− 0.013	0.060***
	(0.006)	(0.015)	(0.007)	(0.012)
Southern Europe G2	0.013	0.038*	0.012	0.044**
	(0.011)	(0.019)	(0.013)	(0.017)
Southern Europe G2.5	0.003	− 0.015	− 0.003	0.036***
	(0.007)	(0.012)	(0.007)	(0.010)
MENA G2	− 0.013**	0.068***	0.016**	0.123***
	(0.004)	(0.014)	(0.005)	(0.011)
MENA G2.5	− 0.015	− 0.006	0.002	0.049**
	(0.008)	(0.018)	(0.009)	(0.015)
Iran G2	0.025*	0.111***	0.067***	0.154***
	(0.010)	(0.022)	(0.014)	(0.019)
Iran G2.5	0.011	0.026	0.021	0.096***
	(0.014)	(0.028)	(0.016)	(0.026)
Turkey G2	− 0.026***	0.079***	− 0.032***	0.079***
	(0.006)	(0.014)	(0.006)	(0.011)
Turkey G2.5	− 0.038**	0.046	− 0.011	0.027
	(0.012)	(0.035)	(0.015)	(0.028)
Other Non-Western G2	− 0.009	0.079***	0.009	0.136***
	(0.005)	(0.012)	(0.006)	(0.011)
Other Non-Western G2.5	− 0.010*	0.050***	0.003	0.070***
	(0.004)	(0.010)	(0.005)	(0.009)

Upper sec. = Upper secondary degrees, Tertiary = Tertiary education degrees. Standard errors in parentheses

*p < 0.05, **p < 0.01, ***p < 0.001

## Discussion and Conclusions

This study explored the overqualification patterns among the second-generation, immigrants, and the majority population, utilising Swedish register data. Using LPMs, I investigated differences in overqualification across immigrant generations and ancestries by gender and educational level. While the G1 reported high probabilities of overqualification, the G2 and the G2.5 displayed similar probabilities of overqualification as the majority population, indicating integration in occupational returns to education in the Swedish labour market. Nevertheless, the G2, especially tertiary-educated women and men, had moderately higher probabilities of overqualification than the majority population. This study also found heterogeneities in overqualification patterns across ancestry groups and educational levels. Among the upper secondary graduates, many G2 groups showed similar or lower probabilities of overqualification compared to their majority population counterparts. However, among individuals with tertiary education degrees, G2 men and women generally reported higher probabilities than the reference group. Also, the gaps between the G2 and the majority population were larger in G2 non-Western groups with tertiary education degrees. While having a native parent was associated with a lower probability of overqualification, G2.5 Finnish and Turkish women with upper secondary degrees stood out as exceptions.

The lower overqualification probabilities observed among the G2 compared to the G1 align with expectations based on the theoretical framework of the determinants for the overqualification of migrants. The lower probability of overqualification among the G2 was expected because the G2 does not encounter the major challenges in education-occupation linkage, such as insufficient host country language proficiency or limited human capital transferability as the G1. This result also corroborates previous findings (Belfi et al., 2022; Fernández-Reino et al., 2018; Larsen et al., 2018). Nevertheless, the persistent gaps in overqualification between the majority population and the G2, even after adjusting for compositional differences, suggest that other determinants—e.g., job search networks, social capital, labour market discrimination, and various forms of unobserved heterogeneity—continue to influence their education-occupation linkage adversely.

The main findings underscore the presence of significant heterogeneity within the G2, which varies by ancestry, educational level, and gender. Non-Western groups consistently report higher probabilities for overqualification, but Nordic groups do not present higher probabilities of overqualification in most cases. This finding suggests that the risk of overqualification is concentrated among origin groups with greater social and cultural distance (Polek et al., 2010). This result also corroborates previous research reporting ethnic penalties in educational mismatch (Falcke et al., 2020). Higher probabilities of overqualification among individuals of non-Western origin may explain the disparity between educational achievements and earnings among certain G2 groups, e.g., G2 Iranian, that previous research documented (Harber-Aschan et al., 2022). This result may be due to heterogeneity across ancestry groups, such as the socio-economic resources of their co-ethnic networks (Bygren & Szulkin, 2010) or ethnic discrimination in hiring (Rafferty, 2020). In addition, differences in socio-economic background and parental social capital may contribute to the observed gaps (Roth & Weißmann, 2022; Zwysen & Longhi, 2018).

The lower probabilities of overqualification among G2.5 individuals, especially with the Eastern European, Iranian, MENA, and Other Non-Western origin groups, may indicate that social capital and the job search network were one of the essential barriers for them to find an adequately matched job. The results also suggest that having more frequent contact with the majority population, which enriches the source of information and widens job search networks, benefits non-Western origin groups more than Western (including Nordic) origin groups. Meanwhile, the reverse relationship for the Finnish and Turkish G2.5 individuals suggests that having mixed backgrounds may adversely affect labour market outcomes, as previous research reported (Loi et al., 2021). However, it takes extra caution to interpret these results, as they may also be influenced by intermarriage (Kalmijn, 2015). Therefore, the observed pattern warrants further investigation into the underlying mechanisms.

Furthermore, perceived social and cultural distances associated with ancestry do not fully explain the results. For example, G2 Finnish and Other Western women with tertiary education degrees and G2 Other Western women and men with upper secondary degrees showed relatively higher overqualification probabilities than the majority Swedish population. Concurrently, G2 MENA, Turkish, and Other Non-Western men and G2 Turkish women with upper secondary degrees had lower or similar probabilities compared to the majority Swedish counterparts. The results for Finnish-origin individuals align with other labour market disadvantages they experience (e.g., Aradhya et al., 2023). However, the Other Western-origin group is generally considered a well-integrated ancestry group in Sweden, whereas the Turkish-origin group is often associated with a high level of labour market disadvantages (Aradhya et al., 2023; Grotti et al., 2023). One explanation is the selection into higher education. As seen in Table 7 in the Appendix, the Other Western group showed a higher proportion of the tertiary educated than other groups. Therefore, those who remained as upper secondary graduates may be more negatively selected regarding unmeasured productivity traits. For MENA, Turkish, and Other Non-Western groups, positive selection into employment due to their higher unemployment risk (Aradhya et al., 2023) may play a role.

Two noteworthy patterns were observed regarding the differences in probabilities of overqualification by gender and education levels. First, although highly educated individuals are more likely to be overqualified in most ancestry groups, there are some exceptions among some origin groups, such as G2 Other Western men and women, Eastern European men, and Southern European men. This pattern was most pronounced among the G2 Other Western group and not expected by previous research. Although the adverse selection of upper secondary graduates may explain their high probabilities of overqualification, more research is needed to explain why a tertiary education degree is associated with lower probabilities than an upper secondary degree. Second, G2 women with tertiary education degrees showed a larger increase in probabilities of overqualification than G2 men with tertiary education degrees. This pattern holds for all ancestry groups except for the G2 Turkish group. This difference may suggest that the allocative discrimination against female employees with immigrant backgrounds (Karakaya et al., 2007) is more pronounced among jobs or occupations that tertiary graduates would generally pursue. If this is the case, this finding provides an opposite case of more substantial labour market discrimination against ethnic minority men in the Swedish context (Arai et al., 2016). However, this study does not provide any further empirical evidence, and future research is needed to explain why the gap between the majority population and the G2 is more significant in highly educated women than men in Sweden.

This study is one of the first studies to examine overqualification patterns of the children of immigrants and to highlight the heterogeneity across ancestry. Nonetheless, this study comes with limitations. First, the comparison between the G1 and the G2 in this study does not compare the immigrants to their children but concurrent G1 and G2 individuals. Thus, the conclusions about integration in occupational returns to education should be taken cautiously. Second, the RM method depends on the distribution of years of schooling for each occupational group, and the choice of cut-off may be arbitrary (Capsada-Munsech, 2019). I attempted to alleviate this issue using modal values rather than mean years of schooling. Third, the Swedish Occupational Register does not cover every worker registered as employed. In the study data, individuals with missing occupations reported lower average labour market earnings and fewer years of education. The proportion of the G1 and the G2 individuals is also higher among those with missing data regarding occupation, which indicates that missing occupation data did not occur randomly. Although not included in the study, the comparison between the year 2014 (lower coverage due to the introduction of SSYK 2012) and year 2016 did not show a clear trend in underestimating or overestimating the prevalence of overqualification across immigrant generations or ancestry groups, suggesting the bias imposed by non-random missingness in the Swedish Occupational Register is limited. Fourth, another issue related to selection is potential over-coverage in population register data. The lack of accurate emigration documentation is a major factor contributing to over-coverage in the Swedish population register (Monti et al., 2020). The degree of over-coverage may vary depending on one’s educational standing. It could introduce bias in the proportion of overqualified individuals, as a recent study indicated that underqualification is positively associated with emigration and overqualification is negatively associated with emigration (Wanner et al., 2021).

The main findings of this study offer two key insights into the overqualification of immigrants and their descendants in high-income host countries. First, G2 individuals continue to face disadvantages in educational mismatches compared to the majority population, particularly those from non-Western backgrounds. This suggests that disparities in earnings, career progression, and other life outcomes between the G2 and the majority population may stem from varying overqualification risks across ancestry groups. Furthermore, those most vulnerable to overqualification—often well-educated and employed—are frequently overlooked in discussions about immigrant integration. Despite being perceived as well-integrated based on these criteria, their significant struggles with education-occupation alignment remain underacknowledged in both academic literature and policy debates. Second, from a policy perspective, individuals with immigrant backgrounds require more support to secure adequate job matches. Their higher risk of overqualification not only limits the returns on their educational investment but also hinders the host country’s ability to fully utilise its human capital, potentially leading to productivity losses (Serikbayeva & Abdulla, 2022).

## Data Availability

Data may be obtained from a third party and are not publicly available. The author can make aggregated data available, conditional on ethical vetting. The author accessed the individual-level data through Statistics Sweden’s micro-online access system MONA. The analyses have been approved by the Swedish ethical vetting authority (Dnr 2017/1980-31/5).

## Appendix

See Tables

**Table 4:** Descriptive statistics by immigrant generation

	Majority	G2	G1	Total
N	2,420,220	343,685	470,840	3,234,745
(%)	77.0	10.2	12.8	100.0
*Gender*				
Men	49.8	49.9	48.9	49.6
Women	50.3	50.1	51.1	50.4
*Educational attainment*				
Upper secondary	61.3	62.9	52.4	60.2
Tertiary	38.7	37.1	47.6	39.8
*Region*				
Rural	18.7	12.1	6.5	16.2
Suburban	9.5	7.2	5.1	8.6
Urban	71.9	80.7	88.5	75.2
Age	43.1	40.9	42.4	42.7
(Std. dev.)	12.2	12.2	11.1	12.0

Majority = the Majority Swedish population, G2 = the second-generation, G1 = the first-generation immigrants

4,

**Table 5:** Descriptive table by ancestry, Swedish-born individuals

	SW	FI	O.N.	O.W.	E.E.	F.Y.	S.E.	M.E.	IR	TU	OT	Total
N	2,420,220	121,425	50,464	49,766	32,616	22,011	13,458	15,607	4,433	10,179	23,726	2,763,905
(%)	87.6	4.4	1.8	1.8	1.2	0.8	0.5	0.6	0.2	0.4	0.9	100.0
*Gender*												
Men	49.8	49.4	49.5	50.2	49.4	50.8	51.1	50.6	52.2	49.0	50.8	49.8
Women	50.3	50.6	50.5	49.8	50.6	49.2	49.0	49.4	47.8	51.0	49.2	50.2
*Educational attainment*												
Upper secondary	61.3	65.9	66.5	55.8	54.7	68.7	58.8	66.6	57.4	64.5	60.8	61.5
Tertiary	38.7	34.1	33.5	44.3	45.4	31.3	41.2	33.5	42.6	35.5	39.2	38.5
*Region*												
Rural	18.7	15.6	17.0	13.8	10.5	5.6	6.4	2.6	2.7	2.4	4.4	17.9
Suburban	9.5	8.4	9.7	7.6	6.8	5.8	5.1	2.7	2.1	1.7	3.7	9.2
Urban	71.9	76.0	73.4	78.6	82.7	88.6	88.5	94.7	95.2	95.9	91.9	73.0
Age	43.1	42.5	45.6	44.8	44.2	35.0	39.0	28.7	27.7	31.0	30.9	42.8
(Std. dev.)	12.2	11.1	12.4	11.6	12.2	9.8	9.9	7.4	5.8	6.9	8.7	12.2

SW = Sweden, FI = Finland, O.N. = Other Nordic, O.W. = Other Wester, E.E. = Eastern Europe, F.Y. = Former Yugoslavia, S.E. = Southern Europe, MENA = Middle East and North Africa, IR = Iran, TU = Turkey, OT = Other Non-Western

5,

**Table 6:** The share of overqualified employees in the study population (%) by immigrant generation status and gender, in 2013 and 2016, using the mean-based and the mode-based methods

	Mode-based				Mean-based			
	Majority	G2	G1	Total	Majority	G2	G1	Total
*2016*								
Men	27.8	27.5	43.3	30.0	12.0	12.2	28.1	14.3
Women	26.7	27.5	42.1	29.1	11.7	12.4	26.6	14.0
*2013*								
Men	28.1	27.7	43.4	30.0	12.7	12.9	27.5	14.6
Women	27.7	28.7	42.8	29.8	12.0	12.6	25.5	13.8

Majority = the Majority Swedish population, G2 = the second-generation, G1 = the first-generation immigrants

6,

**Table 7:** The share of overqualified employees in the study population born in Sweden (%) by ancestry and gender in 2013 and 2016, using the mean-based and the mode-based methods

	SW	FI	O.N.	O.W.	E.E.	F.Y.	S.E.	M.E.	IR	TU	OT	Total
*2016*												
*Mode-based*												
Men	27.8	26.4	26.8	33.2	33.7	23.6	28.9	19.7	26.2	22.0	24.6	27.8
Women	26.7	26.9	26.7	31.9	32.0	24.2	27.9	23.0	28.5	22.4	25.2	26.8
*Mean-based*												
Men	12.0	11.0	11.2	15.3	16.1	10.1	11.9	9.5	15.0	9.7	13.5	12.0
Women	11.7	11.7	11.2	14.3	15.1	11.1	13.0	11.5	15.7	10.2	13.7	11.8
*2013*												
*Mode-based*												
Men	28.1	25.3	27.4	34.0	34.6	24.0	28.2	21.0	25.5	19.7	25.7	28.1
Women	27.7	27.9	28.2	32.4	32.7	26.2	28.1	22.5	29.0	21.5	26.9	27.8
*Mean-based*												
Men	12.7	11.5	11.7	16.0	16.7	11.2	12.9	10.5	13.4	9.8	13.8	12.7
Women	12.0	11.6	11.5	14.5	15.2	11.6	12.4	11.4	15.1	10.3	14.4	12.1

SW = Sweden, FI = Finland, O.N. = Other Nordic, O.W. = Other Wester, E.E. = Eastern Europe, F.Y. = Former Yugoslavia, S.E. = Southern Europe, MENA = Middle East and North Africa, IR = Iran, TU = Turkey, OT = Other Non-Western

7,

**Table 8:** The point estimates of the differences in the probability of overqualification across immigrant generation groups from LPMs, standard errors in parentheses

	(1)	(2)	(3)
	Model1	Model2	Model3
*Immigrant generation*			
*(Reference* = *Majority population)*			
2.5 Generation (G2.5)	0.010***	0.001	0.002**
	(0.001)	(0.001)	(0.001)
Second-generation (G2)	0.025***	0.004*	0.006***
	(0.001)	(0.002)	(0.001)
First-generation (G1)	0.129***	0.057***	0.047***
	(0.001)	(0.001)	(0.001)
*Gender*			
*(Reference* = *Men)*			
Women	− 0.051***	0.001	− 0.012***
	(0.000)	(0.001)	(0.000)
*Educational attainment*			
*(Reference* = *Upper secondary)*			
Tertiary	0.258***	0.303***	0.280***
	(0.001)	(0.001)	(0.001)
Age	0.017***	0.017***	− 0.002***
	(0.000)	(0.000)	(0.000)
Age squared	− 0.000***	− 0.000***	0.000***
	(0.000)	(0.000)	(0.000)
*Region*			
*(Reference* = *Rural)*			
Suburban	− 0.004***	− 0.003**	− 0.004***
	(0.001)	(0.001)	(0.001)
Urban	0.013***	0.014***	− 0.002***
	(0.001)	(0.001)	(0.000)
*Interaction terms*			
G2.5 & Women		0.006**	0.002*
		(0.002)	(0.001)
G2 & Women		0.003	0.000
		(0.003)	(0.001)
G1 & Women		− 0.016***	− 0.013***
		(0.002)	(0.001)
G2.5 & Tertiary		0.005	0.006*
		(0.003)	(0.003)
G2 & Tertiary		0.039***	0.019***
		(0.005)	(0.004)
G1 & Tertiary		0.129***	0.186***
		(0.002)	(0.002)
Women & Tertiary		− 0.137***	− 0.078***
		(0.001)	(0.001)
G2.5 & Women & Tertiary		0.016***	0.011***
		(0.004)	(0.003)
G2 & Women & Tertiary		0.020**	0.016**
		(0.006)	(0.005)
G1 & Women & Tertiary		0.065***	0.022***
		(0.003)	(0.003)
Constant	− 0.241***	− 0.264***	0.056***
	(0.003)	(0.003)	(0.002)
Observations	3,234,745	3,234,745	3,234,745
Adjusted R-squared	0.110	0.118	0.177

^*^*p* < 0.05, ***p* < 0.01, ****p* < 0.001

^8^,

**Table 9:** The point estimates of the differences in the probability of overqualification across ancestry groups from LPMs, standard errors in parentheses

	(1)	(2)	(3)	(4)
	Model4	Model5	Model6	Model7
*Ancestry*				
*(Reference* = *Sweden)*				
Finland	0.010***	0.001	0.010***	0.003
	(0.002)	(0.002)	(0.001)	(0.002)
Other Nordic	0.008**	0.001	0.009***	0.002
	(0.002)	(0.003)	(0.002)	(0.003)
Other Western	0.040***	0.048***	0.016***	0.048***
	(0.003)	(0.004)	(0.002)	(0.004)
East-Eu	0.042***	0.044***	0.016***	0.040***
	(0.003)	(0.004)	(0.002)	(0.004)
Former Yugoslavia	0.018***	− 0.003	0.001	− 0.002
	(0.003)	(0.004)	(0.002)	(0.004)
South-Eu	0.021***	0.015**	0.004	0.015*
	(0.004)	(0.006)	(0.003)	(0.006)
MENA	0.027***	− 0.010**	− 0.003	− 0.013***
	(0.003)	(0.004)	(0.002)	(0.004)
Iran	0.071***	0.025**	0.009	0.030***
	(0.006)	(0.008)	(0.005)	(0.009)
Turkey	0.010*	− 0.026***	− 0.004	− 0.022***
	(0.004)	(0.005)	(0.002)	(0.005)
Other	0.039***	− 0.002	0.005*	0.001
	(0.003)	(0.004)	(0.002)	(0.004)
*G2.5*				
*(Reference* = *No)*				
Yes	− 0.012***	− 0.013***	− 0.010***	− 0.014***
	(0.002)	(0.002)	(0.001)	(0.002)
*Gender*				
*(Reference* = *Men)*				
Women	− 0.049***	0.001*	− 0.012***	0.001*
	(0.001)	(0.001)	(0.000)	(0.001)
*Educational attainment*				
*(Reference* = *Upper secondary)*				
Tertiary	0.231***	0.303***	0.280***	0.303***
	(0.001)	(0.001)	(0.001)	(0.001)
Age	0.017***	0.017***	− 0.003***	0.017***
	(0.000)	(0.000)	(0.000)	(0.000)
Age squared	− 0.000***	− 0.000***	0.000***	− 0.000***
	(0.000)	(0.000)	(0.000)	(0.000)
*Region*				
*(Reference* = *Rural)*				
Suburban	− 0.003**	− 0.004***	− 0.005***	− 0.004***
	(0.001)	(0.001)	(0.001)	(0.001)
Urban	0.017***	0.015***	− 0.001**	0.015***
	(0.001)	(0.001)	(0.000)	(0.001)
*Interaction terms*				
Finland & Women		0.005	− 0.001	0.004
		(0.003)	(0.001)	(0.003)
Other Nordic & Women		0.009*	0.000	0.009*
		(0.004)	(0.002)	(0.004)
Other Western & Women		0.004	− 0.002	0.007
		(0.005)	(0.002)	(0.005)
East-Eu & Women		− 0.007	− 0.006*	− 0.005
		(0.006)	(0.003)	(0.006)
Former Yugoslavia & Women		− 0.005	0.006*	− 0.004
		(0.006)	(0.003)	(0.006)
South-Eu & Women		− 0.005	0.007	− 0.004
		(0.009)	(0.004)	(0.009)
MENA & Women		0.025***	0.016***	0.027***
		(0.006)	(0.003)	(0.006)
Iran & Women		0.029*	0.011	0.028*
		(0.013)	(0.007)	(0.014)
Turkey & Women		− 0.001	0.005	− 0.007
		(0.008)	(0.003)	(0.008)
Other & Women		0.015**	0.013***	0.014**
		(0.005)	(0.003)	(0.005)
Finland & Tertiary		0.019***	0.007	0.017***
		(0.004)	(0.004)	(0.004)
Other Nordic & Tertiary		0.008	0.002	0.007
		(0.007)	(0.006)	(0.007)
Other Western & Tertiary		− 0.039***	0.013*	− 0.039***
		(0.006)	(0.005)	(0.006)
East-Eu & Tertiary		− 0.022**	0.019**	− 0.014
		(0.008)	(0.006)	(0.008)
Former Yugoslavia & Tertiary		0.052***	0.018*	0.051***
		(0.010)	(0.009)	(0.010)
South-Eu & Tertiary		− 0.005	− 0.017	− 0.004
		(0.012)	(0.010)	(0.012)
MENA & Tertiary		0.055***	− 0.011	0.060***
		(0.012)	(0.010)	(0.012)
Iran & Tertiary		0.060**	0.039*	0.037
		(0.019)	(0.017)	(0.020)
Turkey & Tertiary		0.102***	− 0.016	0.102***
		(0.014)	(0.012)	(0.015)
Other & Tertiary		0.071***	0.037***	0.077***
		(0.009)	(0.008)	(0.009)
Women & Tertiary		− 0.137***	− 0.078***	− 0.137***
		(0.001)	(0.001)	(0.001)
Finland & Women & Tertiary		0.006	0.012*	0.009
		(0.006)	(0.005)	(0.006)
Other Nordic & Women & Tertiary		− 0.001	0.009	− 0.001
		(0.009)	(0.007)	(0.009)
Other Western & Women & Tertiary		0.031***	0.009	0.026**
		(0.009)	(0.007)	(0.009)
East-Eu & Women & Tertiary		0.041***	0.019*	0.043***
		(0.010)	(0.008)	(0.010)
Former Yugoslavia & Women & Tertiary		0.038**	0.017	0.037**
		(0.014)	(0.011)	(0.014)
South-Eu & Women & Tertiary		0.043**	0.041**	0.041*
		(0.016)	(0.013)	(0.017)
MENA & Women & Tertiary		0.031*	0.030*	0.023
		(0.015)	(0.013)	(0.016)
Iran & Women & Tertiary		0.024	0.010	0.037
		(0.027)	(0.023)	(0.028)
Turkey & Women & Tertiary		− 0.002	0.018	− 0.003
		(0.019)	(0.015)	(0.019)
Other & Women & Tertiary		0.019	0.002	0.017
		(0.012)	(0.010)	(0.012)
Constant	− 0.244***	− 0.261***	0.071***	− 0.261***
	(0.003)	(0.003)	(0.002)	(0.003)
Observations	2,763,905	2,763,905	2,763,905	2,763,905
Adjusted R-squared	0.085	0.090	0.129	0.090

^*^*p* < 0.05, ***p* < 0.01, ****p* < 0.001

^9^,

**Table 10:** Probability of overqualification by ancestry group (distinguishing the G2 and the G2.5), gender, and educational attainment (from LPM), standard errors in parentheses

	(1)	(2)
	Model8	Model9
*Ancestry group*		
*(Reference* = *Sweden)*		
Finland G2	− 0.000	− 0.005
	(0.002)	(0.003)
Finland G2.5	0.003*	− 0.009***
	(0.001)	(0.002)
Other Nordic G2	0.020***	0.007
	(0.006)	(0.008)
Other Nordic G2.5	− 0.006**	− 0.012***
	(0.002)	(0.003)
Other Western G2	0.047***	0.055***
	(0.006)	(0.009)
Other Western G2.5	0.027***	0.034***
	(0.002)	(0.004)
Eastern Europe G2	0.050***	0.051***
	(0.004)	(0.007)
Eustern Europe G2.5	0.027***	0.028***
	(0.003)	(0.005)
Former Yugoslavia G2	0.019***	− 0.001
	(0.003)	(0.005)
Former Yugoslavia G2.5	0.006	− 0.018**
	(0.005)	(0.006)
Southern Europe G2	0.025***	0.013
	(0.007)	(0.011)
Southern Europe G2.5	0.008	0.003
	(0.004)	(0.007)
MENA G2	0.031***	− 0.013**
	(0.004)	(0.004)
MENA G2.5	0.007	− 0.015
	(0.006)	(0.008)
Iran G2	0.084***	0.025*
	(0.008)	(0.010)
Iran G2.5	0.038***	0.011
	(0.010)	(0.014)
Turkey G2	0.011*	− 0.026***
	(0.004)	(0.006)
Turkey G2.5	− 0.005	− 0.038**
	(0.010)	(0.012)
Other G2	0.045***	− 0.009
	(0.004)	(0.005)
Other G2.5	0.023***	− 0.010*
	(0.003)	(0.004)
Gender		
(Reference = Men)		
Women	− 0.049***	0.001*
	(0.001)	(0.001)
Educational attainment		
(Reference = Upper secondary)		
Tertiary	0.231***	0.303***
	(0.001)	(0.001)
Age	0.017***	0.017***
	(0.000)	(0.000)
Age squared	− 0.000***	− 0.000***
	(0.000)	(0.000)
Region		
(Reference = Rural)		
Suburban	− 0.003**	− 0.004***
	(0.001)	(0.001)
Urban	0.017***	0.015***
	(0.001)	(0.001)
*Interaction terms*		
Finland G2 & Women		− 0.001
		(0.005)
Finland G2.5 & Women		0.008*
		(0.003)
Other Nordic G2 & Women		0.013
		(0.013)
Other Nordic G2.5 & Women		0.008
		(0.004)
Other Western G2.5 & Women		0.004
		(0.005)
Eastern Europe G2 & Women		− 0.003
		(0.011)
Eustern Europe G2.5 & Women		− 0.008
		(0.007)
Former Yugoslavia G2 & Women		− 0.011
		(0.007)
Former Yugoslavia G2.5 & Women		0.004
		(0.009)
Southern Europe G2 & Women		− 0.001
		(0.017)
Southern Europe G2.5 & Women		− 0.006
		(0.010)
MENA G2 & Women		0.028***
		(0.007)
MENA G2.5 & Women		0.017
		(0.012)
Iran G2 & Women		0.042*
		(0.017)
Iran G2.5 & Women		0.010
		(0.021)
Turkey G2 & Women		− 0.006
		(0.008)
Turkey G2.5 & Women		0.027
		(0.019)
Other G2 & Women		0.018*
		(0.008)
Other G2.5 & Women		0.014*
		(0.007)
Finland G2 & Tertiary		0.022*
		(0.009)
Finland G2.5 & Tertiary		0.017***
		(0.005)
Other Nordic G2 & Tertiary		− 0.011
		(0.020)
Other Nordic G2.5 & Tertiary		0.011
		(0.007)
Other Western G2 & Tertiary		− 0.034
		(0.017)
Other Western G2.5 & Tertiary		− 0.040***
		(0.007)
Eastern Europe G2 & Tertiary		− 0.032*
		(0.013)
Eustern Europe G2.5 & Tertiary		− 0.017
		(0.009)
Former Yugoslavia G2 & Tertiary		0.058***
		(0.013)
Former Yugoslavia G2.5 & Tertiary		0.045**
		(0.016)
Southern Europe G2 & Tertiary		0.025
		(0.022)
Southern Europe G2.5 & Tertiary		− 0.018
		(0.014)
MENA G2 & Tertiary		0.081***
		(0.014)
MENA G2.5 & Tertiary		0.008
		(0.019)
Iran G2 & Tertiary		0.086***
		(0.024)
Iran G2.5 & Tertiary		0.015
		(0.031)
Turkey G2 & Tertiary		0.105***
		(0.015)
Turkey G2.5 & Tertiary		0.084*
		(0.037)
Other G2 & Tertiary		0.088***
		(0.014)
Other G2.5 & Tertiary		0.060***
		(0.011)
Women & Tertiary		− 0.137***
		(0.001)
Finland G2 & Women & Tertiary		− 0.003
		(0.011)
Finland G2.5 & Women & Tertiary		0.009
		(0.007)
Other Nordic G2 & Women & Tertiary		0.050
		(0.026)
Other Nordic G2.5 & Women & Tertiary		− 0.008
		(0.009)
Other Western G2 & Women & Tertiary		0.022
		(0.024)
Other Western G2.5 & Women & Tertiary		0.032***
		(0.009)
Eastern Europe G2 & Women & Tertiary		0.051**
		(0.018)
Eustern Europe G2.5 & Women & Tertiary		0.036**
		(0.013)
Former Yugoslavia G2 & Women & Tertiary		0.043*
		(0.017)
Former Yugoslavia G2.5 & Women & Tertiary		0.028
		(0.022)
Southern Europe G2 & Women & Tertiary		0.007
		(0.030)
Southern Europe G2.5 & Women & Tertiary		0.057**
		(0.019)
MENA G2 & Women & Tertiary		0.026
		(0.019)
MENA G2.5 & Women & Tertiary		0.038
		(0.026)
Iran G2 & Women & Tertiary		0.001
		(0.034)
Iran G2.5 & Women & Tertiary		0.060
		(0.044)
Turkey G2 & Women & Tertiary		0.006
		(0.020)
Turkey G2.5 & Women & Tertiary		− 0.046
		(0.048)
Other G2 & Women & Tertiary		0.039*
		(0.019)
Other G2.5 & Women & Tertiary		0.006
		(0.015)
Constant	− 0.244***	− 0.262***
	(0.003)	(0.003)
Observations	2,763,905	2,763,905
Adjusted R-squared	0.085	0.090

^*^*p* < 0.05, ***p* < 0.01, ****p* < 0.001

^10^ and

**Table 11:** Country of Ancestral origin groups

	Finland	Other Nordic	Other Western	Eastern European	Former Yugoslavia	Southern European	MENA	Iran	Turkey	Other
Finland	X									
Denmark		X								
Iceland		X								
Norway		X								
UK and Ireland			X							
Germanic states			X							
Netherlands			X							
France and Benelux			X							
USA and Canada			X							
NZ and Australia			X							
Poland				X						
Latvia and Lithuania				X						
East Europe				X						
Bulgaria				X						
Romania				X						
Czech R and Slovakia				X						
Hungary				X						
Estonia				X						
Bosnia Herzegovina					X					
Yugoslavia					X					
South Europe						X				
Greece and Cyprus						X				
Italy and Malta						X				
Somalia and Djibouti							X			
Eritrea							X			
Ethiopia							X			
North Africa (except Egypt)							X			
Egypt							X			
Other Middle East							X			
Lebanon							X			
Syria							X			
Iraq							X			
Afghanistan							X			
Iran								X		
Turkey									X	
Central America and Caribbean										X
Chile										X
South America										X
Other Africa										X
China (excluding Taiwan and HK)										X
Other East Asia										X
Other South-East Asia and Pacific										X
Philippines										X
Vietnam										X
Thailand										X
Pakistan and Bangladesh										X
India Nepal Bhutan										X
Sri Lanka										X
North and South Korea										X
Brazil										X
Other										X

11.

## Footnotes

1 For instance, previous research discussed how stringent employment protection legislation is associated with a “low-skill, low-technology trap” (Di Pietro, 2002, p. 886), or the increased demand for highly-skilled workers due to the skill-biased technical changes (Croce & Ghignoni, 2012).

2 The dataset for the Migrant Trajectories project contains various Swedish registers such as the Total Population Register, the Multi-Generation Register, and the Swedish Longitudinal Integrated Database for Health Insurance and Labour Market Studies.

3 Mother's country of birth was used to identify an individual’s ancestry if the father’s country of birth was missing or the father was Swedish-born.
